# Recent Advances in Protein Homology Detection Propelled by Inter-Residue Interaction Map Threading

**DOI:** 10.3389/fmolb.2021.643752

**Published:** 2021-05-11

**Authors:** Sutanu Bhattacharya, Rahmatullah Roche, Md Hossain Shuvo, Debswapna Bhattacharya

**Affiliations:** ^1^ Department of Computer Science and Software Engineering, Auburn University, Auburn, AL, United States; ^2^ Department of Biological Sciences, Auburn University, Auburn, AL, United States

**Keywords:** protein homology, inter-residue interaction map, protein threading, homology modeling, protein structure prediction

## Abstract

Sequence-based protein homology detection has emerged as one of the most sensitive and accurate approaches to protein structure prediction. Despite the success, homology detection remains very challenging for weakly homologous proteins with divergent evolutionary profile. Very recently, deep neural network architectures have shown promising progress in mining the coevolutionary signal encoded in multiple sequence alignments, leading to reasonably accurate estimation of inter-residue interaction maps, which serve as a rich source of additional information for improved homology detection. Here, we summarize the latest developments in protein homology detection driven by inter-residue interaction map threading. We highlight the emerging trends in distant-homology protein threading through the alignment of predicted interaction maps at various granularities ranging from binary contact maps to finer-grained distance and orientation maps as well as their combination. We also discuss some of the current limitations and possible future avenues to further enhance the sensitivity of protein homology detection.

## Introduction

The development of computational approaches for accurately predicting the protein three-dimensional (3D) structure directly from the sequence information is of central importance in structural biology (Jones et al., 1992; Baker and Sali, 2001; Dill and MacCallum, 2012). While *ab initio* modeling aims to predict the 3D structure purely from the sequence information (Marks et al., 2011; Adhikari et al., 2015; Wang et al., 2016; Adhikari and Cheng, 2018; Greener et al., 2019; Senior et al., 2019; Xu, 2019; Yang et al., 2020; Roche et al., 2021), many protein targets have evolutionary-related (homologous) structures, also known as homologous templates, already available in the Protein Data Bank (PDB) (Berman et al., 2000). Correctly identifying these templates given the sequence of a query protein and building 3D models by performing query–template alignment, a technique broadly known as homology modeling (Altschul et al., 1997; Xu et al., 2003; Wu and Zhang, 2008; Lobley et al., 2009; Wu and Zhang, 2010; Källberg et al., 2012; Ma et al., 2014) often results in highly accurate predicted structural models (Abeln et al., 2017). As such, the success of homology modeling critically depends on the ability to identify the closely homologous template on the basis of sequence similarity and generate accurate query–template alignment. Intuitively, the performance of these methods sharply deteriorates when the direct evolutionary relationship between the query and templates becomes very low, typically when the sequence similarity falls below 30%, the so-called distant-homology modeling scenarios (Bowie et al., 1991; Petrey and Honig, 2005). Protein threading, the most widely used distant-homology modeling technique, aims to address the challenge by leveraging multiple sources of information by mining the evolutionary profile of the query and templates to reveal potential distant homology and perform distant-homology modeling to predict the 3D structure of the query protein.

Existing threading methods exploit a wide range of techniques ranging from dynamic programming to profile-based comparison to machine learning (Jones, 1999; Rychlewski et al., 2000; Xu and Xu, 2000; Skolnick and Kihara, 2001; Ginalski et al., 2003; Marti et al., 2004; Jaroszewski et al., 2005; Söding, 2005; Zhou and Zhou, 2005; Cheng and Baldi, 2006; Peng and Xu, 2009; Lee and Skolnick, 2010; Peng and Xu, 2010; Yang et al., 2011; Ma et al., 2012; Ma et al., 2013; Gniewek et al., 2014). The recent advancement in predicting the inter-residue interaction maps using sequence coevolution and deep learning (Morcos et al., 2011; He et al., 2017; Wang et al., 2017; Adhikari et al., 2018; Hanson et al., 2018; Kandathil et al., 2019; Yang et al., 2020) has opened new possibilities to further improve the sensitivity of distant-homology protein threading by incorporating the predicted inter-residue interaction information. Fueled by this, several efforts have been made in the recent past to integrate interaction maps into threading. For instance, EigenTHREADER (Buchan and Jones, 2017), map_align (Ovchinnikov et al., 2017), CEthreader (Zheng et al., 2019a), CATHER (Du et al., 2020), and ThreaderAI (Zhang and Shen, 2020) have utilized predicted contact maps in protein threading. DeepThreader (Zhu et al., 2018) has exploited finer-grained distance maps for query proteins instead of using binary contacts to improve threading template selection and alignment. DisCovER (Bhattacharya et al., 2020) goes one step further by incorporating inter-residue orientation along with distance information together with topological network neighborhood (Chen et al., 2019) of query–template alignment to further improve threading performance. Here, we provide an overview of the latest advances in protein homology detection propelled by inter-residue interaction map threading.

## Granularities of Protein Inter-Residue Interaction Maps

Protein inter-residue interaction maps are predicted at various resolutions ranging from binary contact maps to finer-grained distance and orientation maps as well as their combination. A low-resolution version of inter-residue interaction is a contact map, which is a square, symmetric matrix with binary entries, where a contact indicates the spatial proximity of a residue pair at a given cutoff distance, typically set to 8Å between the C_α_ or C_β_ carbons of the interacting residue pairs. Inter-residue distance map is finer-grained in that it captures the distribution of real-valued inter-residue spatial proximity information rather than the binary contacts at a fixed cutoff distance. Recent studies (Xu and Wang, 2019; Xu, 2019) have demonstrated the advantage of using distance maps in protein structure prediction over binary contacts as distances carry more physical constraint information of protein structures than contacts. The granularities of predicted distance maps vary from distance histograms to real-valued distances (Greener et al., 2019; Adhikari, 2020; Ding and Gong, 2020; Li and Xu, 2020; Wu et al., 2021; Yang et al., 2020). Very recently, trRosetta (Yang et al., 2020) has introduced inter-residue orientations in addition to distances to capture not only the spatial proximity information of the interacting pairs but also their relative angles and dihedrals. Collectively, inter-residue distances and orientations encapsulate the spatial positioning of the interacting pairs much better than only distances let alone binary contacts.

## Inter-Residue Interaction Map Threading

**FIGURE 1:**
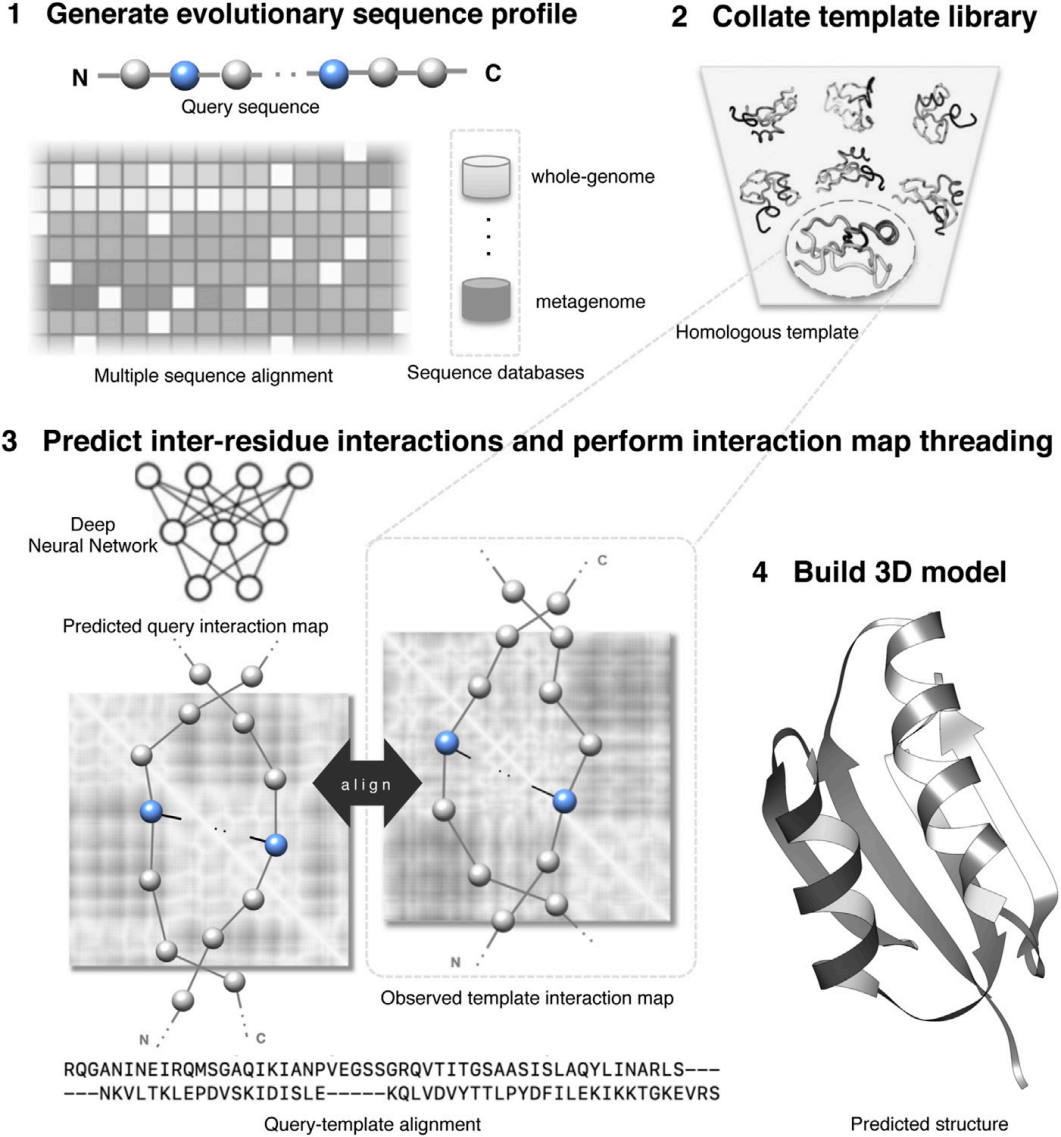
Illustration of protein interaction map threading.

Figure 1 shows an overview of an interaction map threading of a query protein. Generally, threading has four components: (1) an effective scoring function to evaluate the fitness of query–template alignment; (2) efficient template searching or homology detection strategy; (3) optimal query–template alignments; and (4) building 3D models of query proteins based on alignments. One of the most important components of threading approaches is the scoring function, which is composed of standard threading features ranging from sequential features such as secondary structures, solvent accessibility, and sequence profiles to nonlinear features such as pairwise potentials (Bienkowska and Lathrop, 2005; Brylinski and Skolnick, 2010). Weights control the relative importance of different terms. An efficient scoring function should reliably differentiate a homologous template from the alternatives because the accuracy of the predicted model significantly depends on the evolutionary relatedness of the identified template. The inter-residue interaction map helps to improve the sensitivity of the threading scoring function by augmenting the standard scoring terms with additional contributions from the predicted interactions. Specifically, the score to align the i th residue of the query protein to the j th residue of the template can be defined as:

$$E\left(i,j\right)=w_{1}E_{map}^{interaction}\left(i,j\right)+\underset{\begin{array}{c} k\in standard\;\\ threading\;features \end{array}}{\sum }w_{k}E_{k}^{feature}\left(i,j\right)$$

where the first term accounts for the contribution of the interaction map and the second term accounts for the standard threading features with $w_{i}$ being their relative weights. Typically, the similarity between the predicted inter-residue interaction map of the query protein and that derived from the template structure informs the interaction map term in the threading scoring function. It is worth noting here that the raw alignment score is biased to protein length (Xu et al., 2003). As such, most threading methods use a normalized alignment score in standard deviation units relative to the mean score of all templates in the template library for homology detection—detecting best-fit templates from the PDB.

## Emerging Trends in Protein Homology Detection by Interaction Map Threading

With the recent advancement in contact prediction mediated by sequence coevolution and deep learning, significant research efforts have been made in the recent past to incorporate contact information as an additional scoring term into the threading scoring function for protein homology detection. For instance, Jones and coworkers developed EigenTHREADER (Buchan and Jones, 2017) that uses eigen-decomposition (Di Lena et al., 2010) of contact maps predicted using classical neural network–based predictor MetaPSICOV (Jones et al., 2015) to search a library of template contact maps for contact map threading. Baker and coworkers developed map_align (Ovchinnikov et al., 2017) that employs an iterative double dynamic programming framework (Taylor, 1999) for homology detection. map_align takes advantage of metagenomics sequence databases of microbial DNA (Söding, 2017) and uses contact maps predicted by coevolutionary contact predictor GREMLIN (Balakrishnan et al., 2011; Kamisetty et al., 2013) to perform contact map threading by maximizing the number of overlapping contacts and minimizing the number of gaps. Recently, Zhang and coworkers developed CEthreader (Zheng et al., 2019a) using contact maps predicted by deep learning–based contact map predictor ResPRE (Li et al., 2019). CEthreader also relies on eigen-decomposition and performs contact map threading through dynamic programming using a dot-product scoring function by integrating contacts as well as secondary structures and sequence profiles. Alongside, we developed a contact-assisted threading method (Bhattacharya and Bhattacharya, 2019) that incorporates contact information, predicted by deep learning–based predictor RaptorX (Wang et al., 2017), into threading using a two-stage approach. After selecting a subset of top templates from the template library using a standard profile-based threading technique in the first stage, our method subsequently uses eigen-decomposition of the contact information along with the profile-based alignment score to select the best-fit template. We further analyze the impact of contact map quality on threading performance (Bhattacharya and Bhattacharya, 2020), which reveals that incorporating high-quality contact maps having the Matthews correlation coefficient (MCC) ≥ 0.5 improves the threading performance for ∼ 30% cases in comparison to a baseline contact-free threading used as a control, while incorporating low-quality contacts with MCC <0.35 deteriorates the performance for 50% cases. Yang and coworkers developed CATHER (Du et al., 2020) by incorporating contact maps predicted by deep learning–based predictor MapPred (Wu et al., 2020) along with standard sequential information in the threading scoring function. Very recently, Shen and coworkers have developed ThreaderAI (Zhang and Shen, 2020) that implements a neural network for predicting alignments by incorporating deep learning–based contact information with conventional sequential and structural features into the scoring function.

Building on the successes of contact-assisted threading methods, Xu and coworkers developed a distance-based threading method called DeepThreader (Zhu et al., 2018). The method predicts distance maps by employing deep learning and then incorporates the predicted inter-residue distance information along with sequential features into threading through alternating direction method of multipliers (ADMM) algorithm. The inter-residue distance is binned into 12 bins: <5Å, 5–6Å, .., 14–15Å, and >15Å. Based on their reported results as well as the performance evaluation in the 13th Critical Assessment of protein Structure Prediction (CASP13), incorporating distance information boosts threading performance, particularly for distant-homology targets, outperforming contact-assisted threading methods by a large margin (Xu and Wang, 2019, 13). Zhang and coworkers have recently extended CEthreader to develop a distance-assisted threading method DEthreader introduced during the recently concluded CASP14 experiment by incorporating a distance-based scoring term into the scoring function. The method uses the C_α_–C_α_ and C_β_–C_β_ distance distribution, both are binned into 38 bins: 1 bin of <2Å, 36 bins of 2–20Å with a width of 0.5Å, and 1 bin of ≥20Å. Similarly, Yang and coworkers have extended CATHER into a distance-based threading approach by replacing contacts with distances in CASP14.

**FIGURE 2:**
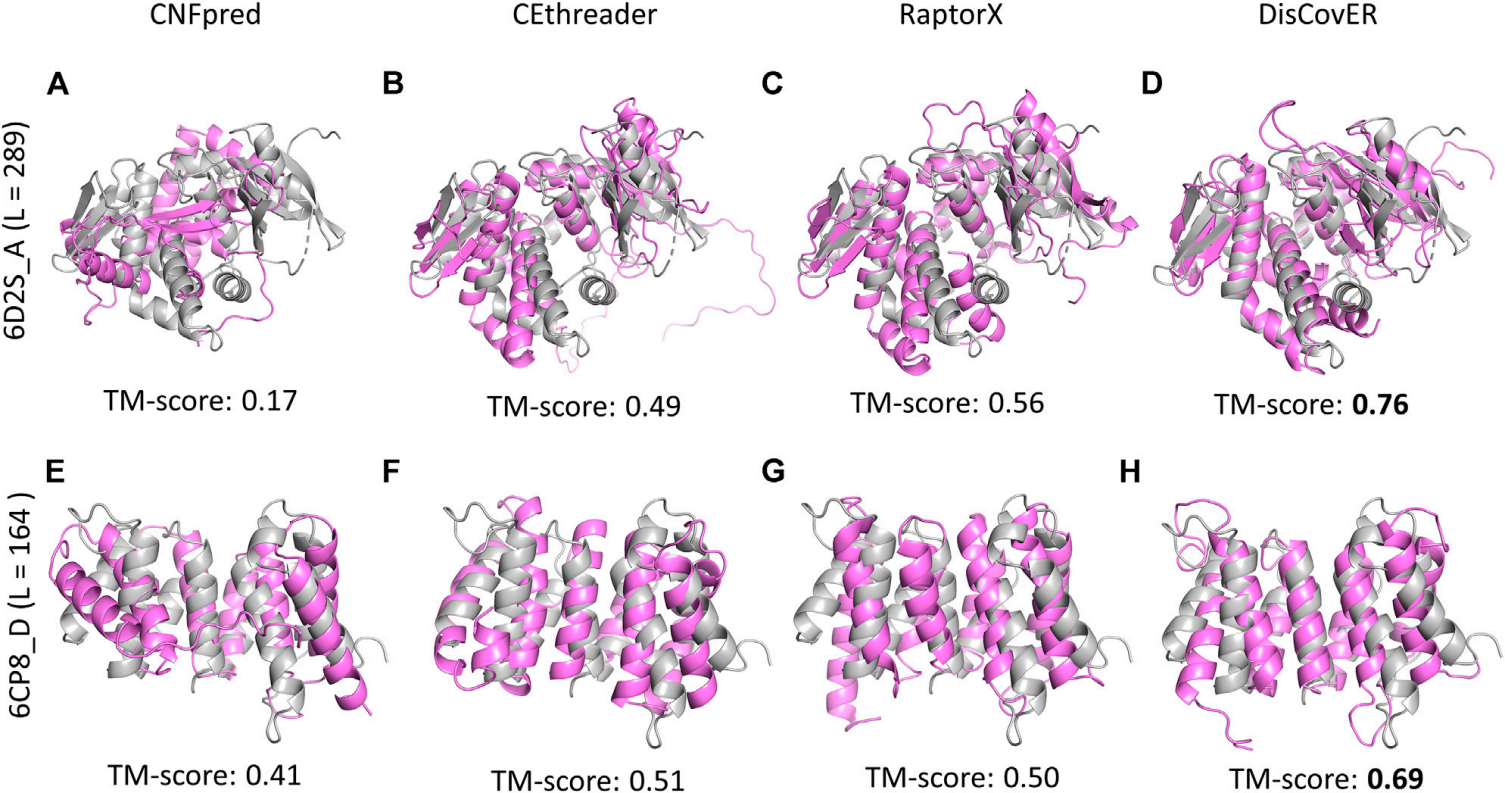
Structural superposition between predicted models using various threading methods (in violet) and the corresponding experimental structures (in gray) for representative CAMEO targets 6D2S_A of length 289 residues and 6CP8_D of length 164 residues.

Powered by the development of the recent deep learning–based trRosetta method (Yang et al., 2020) for the prediction of inter-residue orientations and distances, our recent method DisCovER (Bhattacharya et al., 2020) goes one step further by incorporating predicted inter-residue orientations in addition to distances together with the neighborhood effect of the query–template alignment using an iterative double dynamic programming framework. The predicted distances are binned into 9 bins with a bin size of 1Å: <6Å to <14Å by summing up the likelihoods for distance bins below a distance threshold. The two orientation dihedrals (ω, θ) are binned into 24 bins with a width of 15°, and the orientation angle (ϕ) is binned into 12 bins with a width of 15°. Experimental results demonstrate the improved threading performance of DisCovER over the other state-of-the-art threading approaches on multiple benchmark datasets across various target categories, especially for distantly homologous proteins. Representative examples on CAMEO targets 6D2S_A and 6CP8_D provide some insights into the origin of the improved performance. Figure 2 shows our recent method DisCovER predicts correct folds (TM-score > 0.5) for both the targets 6D2S_A and 6CP8_D with a TM-score of 0.76 and 0.69, respectively, significantly better than the others. While the pure profile-based threading method CNFpred (Ma et al., 2012; Ma et al., 2013) and the recent contact-assisted threading method CEthreader fail to predict the correct fold for the target 6D2S_A, DisCovER and the CAMEO server RaptorX (Källberg et al., 2012; Zhu et al., 2018), employing the distance-based threading method DeepThreader (Haas et al., 2019), effectively predict the correct fold, with noticeably better performance by DisCovER (an improvement of 0.2 TM-score points) than the next best RaptorX. We also notice the superior performance of DisCovER for the target 6CP8_D where DisCovER significantly outperforms the other competing methods including the next best CEthreader by 0.18 TM-score points. It is worth mentioning both the targets are officially classified as “hard” by CAMEO (Haas et al., 2019), which warrants a distantly homologous nature in which current threading methods have limitations. Overall, the results show that the integration of the orientation information and the neighborhood effect in DisCovER results in improved threading, attaining state-of-the-art performance in (distant) homology detection.

## The Role of Sequence Databases in Interaction Map Threading

The prediction of inter-residue interaction maps depends heavily on the availability of homologous sequences. As such, the role of the sequence databases is becoming increasingly important in protein homology detection via interaction map threading. In addition to the well-established whole-genome sequence databases such as the nr database from the National Center for Biotechnology Information (NCBI), UniRef (Suzek et al., 2015), UniProt (The UniProt Consortium, 2019), and Uniclust (Mirdita et al., 2017); emerging metagenome sequence databases from the European Bioinformatics Institute (EBI) Metagenomics (Markowitz et al., 2014; Mitchell et al., 2018) and Metaclust (Steinegger and Söding, 2018) are playing a prominent role. For example, Wang et al. (2019) have demonstrated the applications of marine metagenomics for improved protein structure prediction. map_align uses the Integrated Microbial Genomes (IMG) database (Markowitz et al., 2014), containing around 4 million unique protein sequences, to reliably predict high-quality models for distant-homology Pfam families of unknown structures. Another recent method for generating protein multiple sequence alignments, DeepMSA (Zhang et al., 2020), combines whole-genome and metagenome sequence databases and reports improved threading performance, particularly for distant-homology proteins. Newer sequence databases are getting larger and diverse. For example, BFD (Steinegger et al., 2019), a recent sequence database, is one of the largest sequence databases containing 2 billion protein sequences from soil samples and 292 million sequences of marine samples. Another very recent sequence database MGnify (Mitchell et al., 2020) contains around 1 billion nonredundant protein sequences. As such, the availability of evolutionary information of distant-homology proteins is getting enriched, likely leading to improved prediction accuracy of inter-residue interaction maps and hence more accurate interaction map threading for distant-homology protein modeling.

## Discussion

While the use of interaction maps is the main driving force behind the improved threading performance, the optimal granularity and information content of the predicted interaction maps remain elusive. Existing works consider various distance bins (Zhu et al., 2018; Bhattacharya et al., 2020) and subsets of predicted interactions either based on top predicted pairs sorted based on their likelihood values or using arbitrary likelihood cutoffs (Bhattacharya and Bhattacharya, 2019; Zheng et al., 2019a). A robust mechanism for defining and selecting interacting residue pairs can be beneficial to existing threading methods. Another challenge is how to integrate heterogeneous sources of available information from multiple interaction map predictors and/or sequence databases in a singular framework for unified interaction map threading. Finally, the use of multiple templates (Cheng, 2008; Peng and Xu, 2011; Meier and Söding, 2015) and meta-approaches (Wu and Zhang, 2007; Zheng et al., 2019b) possibly coupled with model quality assessment methods (Ray et al., 2012; Uziela et al., 2016; Uziela et al., 2017; 3; Alapati and Bhattacharya, 2018; Karasikov et al., 2019; Baldassarre et al., 2020; Eismann et al., 2020; Shuvo et al., 2020) and potentially aided by structure refinement (Bhattacharya and Cheng, 2013a; Bhattacharya and Cheng, 2013b; Bhattacharya and Cheng, 2013c; Bhattacharya et al., 2016; Bhattacharya, 2019; Wang et al., 2020; Heo and Feig, 2020) can collectively improve the accuracy of distant-homology protein modeling.

Recent CASP experiments have witnessed dramatic recent advances by DeepMind’s AlphaFold series (Senior et al., 2019; Senior et al., 2020) in *ab initio* protein structure prediction, significantly outperforming the other groups. The success of AlphaFold series is primarily attributed to the successful application of deep neural networks for accurately predicting inter-residue spatial proximity information coupled with end-to-end training, significantly improving the accuracy of protein structure prediction (Pearce and Zhang, 2021). The integration of deep learning into various stages of protein modeling represents an exciting future direction that shall have a transformative impact on distant-homology protein modeling via interaction map threading, complementing and supplementing *ab initio* protein structure prediction methods developed by DeepMind.
